# Genetic Liability to Disability Pension in Women and Men: A Prospective Population-Based Twin Study

**DOI:** 10.1371/journal.pone.0023143

**Published:** 2011-08-05

**Authors:** Jurgita Narusyte, Annina Ropponen, Karri Silventoinen, Kristina Alexanderson, Jaakko Kaprio, Åsa Samuelsson, Pia Svedberg

**Affiliations:** 1 Division of Insurance Medicine, Department of Clinical Neuroscience, Karolinska Institutet, Stockholm, Sweden; 2 Institute of Biomedicine, University of Eastern Finland, Kuopio, Finland; 3 Population Research Unit, Department of Social Research, University of Helsinki, Helsinki, Finland; 4 Department of Public Health, University of Helsinki, Helsinki, Finland; 5 Institute for Molecular Medicine, University of Helsinki, Helsinki, Finland; 6 Department of Mental Health and Substance Abuse Services, National Institute for Health and Welfare, Helsinki, Finland; University of Hong Kong, Hong Kong

## Abstract

**Background:**

Previous studies of risk factors for disability pension (DP) have mainly focused on psychosocial, or environmental, factors, while the relative importance of genetic effects has been less studied. Sex differences in biological mechanisms have not been investigated at all.

**Methods:**

The study sample included 46,454 Swedish twins, consisting of 23,227 complete twin pairs, born 1928–1958, who were followed during 1993–2008. Data on DP, including diagnoses, were obtained from the National Social Insurance Agency. Within-pair similarity in liability to DP was assessed by calculating intraclass correlations. Genetic and environmental influences on liability to DP were estimated by applying discrete-time frailty modeling.

**Results:**

During follow-up, 7,669 individuals were granted DP (18.8% women and 14.1% men). Intraclass correlations were generally higher in MZ pairs than DZ pairs, while DZ same-sexed pairs were more similar than opposite-sexed pairs. The best-fitting model indicated that genetic factors contributed 49% (95% CI: 39–59) to the variance in DP due to mental diagnoses, 35% (95% CI: 29–41) due to musculoskeletal diagnoses, and 27% (95% CI: 20–33) due to all other diagnoses. In both sexes, genetic effects common to all ages explained one-third, whereas age-specific factors almost two-thirds, of the total variance in liability to DP irrespective of diagnosis. Sex differences in liability to DP were indicated, in that partly different sets of genes were found to operate in women and men, even though the magnitude of genetic variance explained was equal for both sexes.

**Conclusions:**

The findings of the study suggest that genetic effects are important for liability to DP due to different diagnoses. Moreover, genetic contributions to liability to DP tend to differ between women and men, even though the overall relative contribution of genetic influences does not differ by sex. Hence, the pathways leading to DP might differ between women and men.

## Introduction

Disability pension (DP) is the ultimate consequence of permanent incapacity to work on medical grounds. It is not only a severe consequence for the individual in terms of, for example, income loss, but also for employers and society [1]. Previous studies of DP have mainly focused on work environmental and psychosocial predictors [2], although pathways leading to DP may also be influenced by biological, including genetic, factors [3]. Research on the genetic and environmental mechanisms involved in the development of DP is scarce [4], and possible sex differences in the genetic component of liability to DP have not been investigated at all.

Musculoskeletal and psychiatric diagnoses jointly account for about 70% of all granted DPs [5]. Previous twin and family studies suggest that genetic factors explain a moderate to a large extent of the variation in most chronic diseases. It is estimated that heritability, the proportion of variance accounted for by genetic factors, is important in conditions underlying the processes leading to DP, such as low back pain (30%), rheumatoid arthritis (60%), depression (40%), anxiety (20%), and hypertension (50%) [6], [7], [8], [9], [10]. These findings suggest that genetic effects may contribute to liability to DP. However, in addition to genetic contributions to a specific disease, liability to DP may also be influenced by genetic effects that account for the variance in other risk factors for DP. These factors include, for example, functional ability, birth weight, and presence or absence of deviant behavior [3], [11], and are primarily indicators of general developmental pathways to health or disease.

Sex differences in DP have been widely investigated, and a higher incidence of DP has been reported among women compared with men [3], [12], [13], [14]. It is also known that demographic, socio-economic, and work-related factors differ between the sexes in relation to future DP [2], [13], [15], [16]. In contrast, both sexes have shown similar levels of risk of DP that emanate from biological and social background factors in childhood [3]. Despite attempts in several studies to identify the impacts of several predictors of DP among women and men, only a few have investigated possible sex differences in pathways leading to DP [3].

To our knowledge, only one study has investigated the importance of genetic and environmental factors for DP, using a 30-year follow-up of Finnish twins [4]. The results showed that liability to DP at any age, due to a variety of diagnoses, was moderately explained by genetic factors (24–48%). The highest genetic variance was observed in liability to DP due to cardiovascular diagnoses (48%), whereas heritable factors were of less importance for variance in liability to DP from all other diagnoses (24%). However, possible sex differences in liability to DP could not be tested in that study, both because of the modest number of DPs granted during the follow-up period and due to a shortage of opposite-sex (OS) twins. Including OS twins not only permits evaluation of whether there are sex differences in the relative importance of genetic influences, but also provides an opportunity to test whether the sets of genes operating in the two sexes differ. In this study, the aims were to investigate: (1) the importance of genetic and environmental factors for liability to DP due to the main DP diagnoses (mental and musculoskeletal) by following a large Swedish population-based twin sample over 16 years, and (2) whether the etiology of liability to DP differs between women and men.

## Methods

### Ethics statement

The study was approved by the Stockholm Regional Ethical Review Board in Sweden, Dnr: 2007/524-31, 2007-05-08 and 2009-08-25.

### Sample

The data come from a large population-based prospective Swedish twin study, the Swedish Twin Study of Disability Pension and Sickness Absence (STODS), which includes all twins from the Swedish Twin Registry (STR) born in Sweden between 1925 and 1958 (n = 59,598 individuals) [17], [18]. Approximately one-third of all the twins are monozygotic (MZ), one-third are same-sexed dizygotic (DZ), and one-third are OS DZ twins. Assignment of zygosity was based on questions about twin intra-pair similarity in childhood. This method was validated with DNA, and showed 99% or higher accuracy [17].

The study sample included all individuals that were alive, living in Sweden and at risk of DP on January 1^st^ 1993. Individuals who were no longer at risk of DP were older than 65, had emigrated, taken old-age retirement, or were on DP before or on January 1^st^ 1993. Twin pairs with unknown zygosity or with information available for only one co-twin (i.e., information on DP, death, emigration, or old-age retirement was missing for one of the twins) were excluded from the data analyses. Thus, the final study sample included 46,454 twin individuals, whereof 2,547 male MZ, 4,164 male DZ, 3,001 female MZ and 4,380 female DZ twin pairs, and 9,135 OS twin pairs. The individuals were followed from January 1^st^ 1993 to the date of DP, old-age retirement, emigration, age of 65, death, or to the last day of the follow-up, December 31^st^ 2008. Thus, the individuals eligible for DP during follow-up were between 34 and 64 years-old.

In Sweden, any person younger than 65 years of age with a medically confirmed disease or injury that has led to permanent work incapacity can be granted DP. For all twins, data on date of and main diagnosis for DP were obtained from the National Social Insurance Agency for the years 1993–2008. DP diagnoses were based on the 9^th^ and 10^th^ revisions of the International Classification of Diseases (ICD) [19]. For the purposes of this study, ICD-9 diagnoses were re-coded to their ICD-10 equivalents. Mental disorders were diagnosed using ICD-10 sections F00-F99, and musculoskeletal disorders encompassed ICD-10 sections M00-M99.

Data on old-age retirement and migration were acquired from Statistics Sweden, and on deaths during the follow-up from the National Board of Health and Welfare. All registry data were linked to the twins by using the unique ten-digit personal identification number assigned to all Swedish residents.

### Statistical analyses

Cumulative incidence rates of DP for each zygosity, sex, and diagnosis groups were calculated for all individuals. Incidence rates of DPs per 1000 person-years and numbers of concordant and discordant twin pairs were computed by age and sex. The first indication of whether genetic or environmental factors are important for liability to DP can be obtained by comparing how similar (i.e., concordant) MZ and DZ twin pairs are for DP. Within-pair similarity for liability to DP was assessed by calculating intraclass tetrachoric correlations for each zygosity, sex, and diagnosis group, and also within age groups. Descriptive statistics and intraclass correlations were computed using SAS statistical software [20].

Genetic and environmental influences on liability to DP were estimated by applying discrete-time frailty models of DP due to all diagnoses, and separately for DP due to mental diagnoses, musculoskeletal diagnoses, and other diagnoses. Discrete-time frailty modeling is an extension of discrete-time survival analysis using a general latent variable framework, which allows for description of the relationship between hazard and latent factors [21], [22]. In this study, the probit of the hazard function was modeled as a function of discretized age at onset of DP and latent genetic and environmental factors [4]. A detailed description of the discrete-time frailty model is provided elsewhere [4], [22].

**Figure 1:**
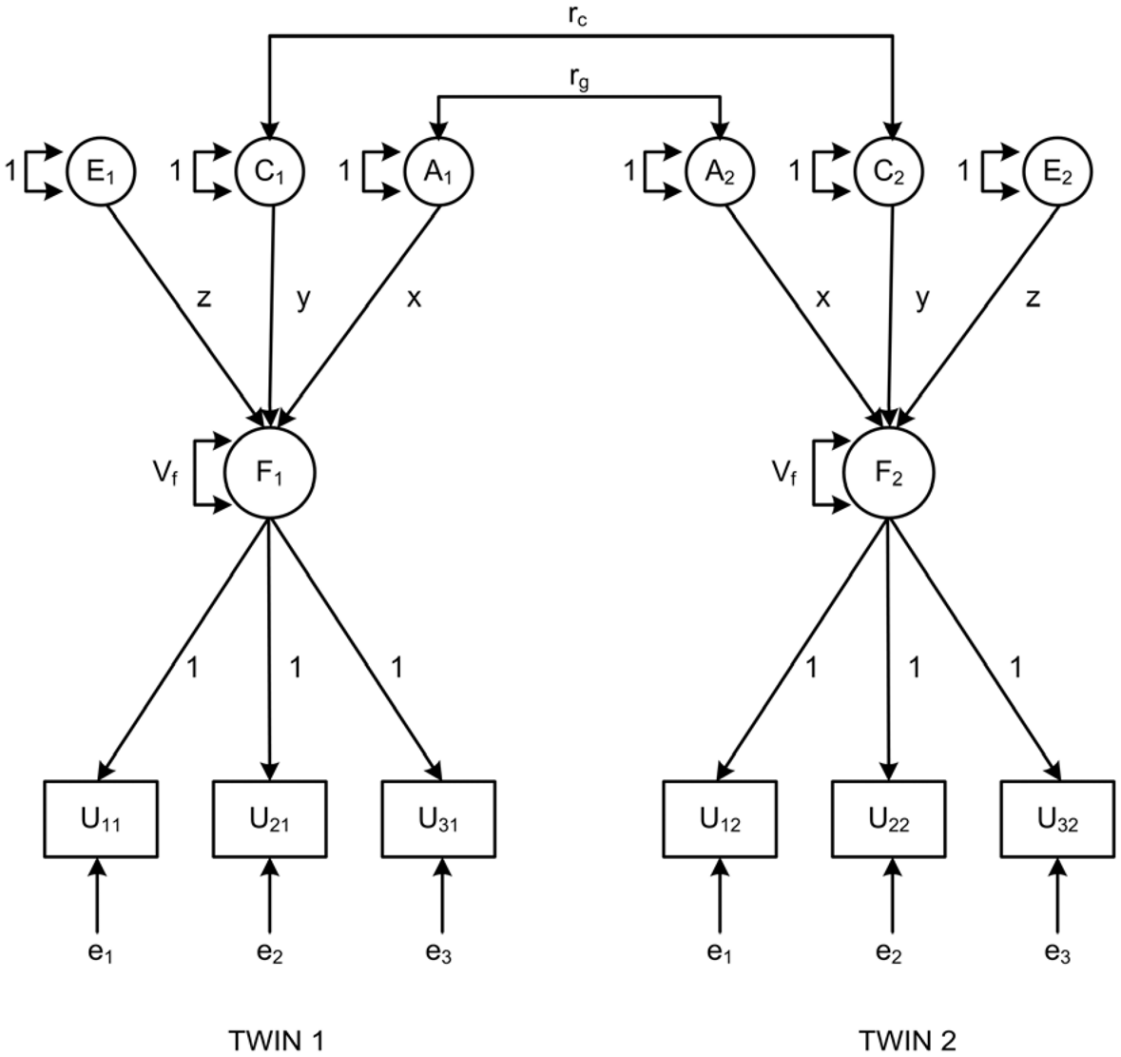
Structural equation model of discrete-time frailty for disability pension (DP) at three age intervals (≤49, 50–57, and 58–64 years). Observed phenotypes, U_ti_, are denoted by rectangles and are the ages at which DP was granted during follow-up. *t* stands for age interval (t = 1, 2, 3 corresponding to ≤49, 50–57, and 58–64 years) and *i* for twin in a pair (i = 1, 2). Unobserved, or latent, genetic (A), shared environmental (C), and unique environmental (E) influences are denoted by circles. Unobserved, or latent, frailty (F_i_) is depicted by a circle, and is common to specific age intervals. The factor loadings of the age intervals (e_t_) are set at 1. The variance in latent frailty, V_f_, can be divided into additive genetic (A), shared environmental (C), and unique environmental (E) variance components. Path coefficients x, y, and z stand for the standard deviations of the estimated variance components, A, C, and E, respectively. The estimates of variance components are defined as: a) heritability h^2^ = x^2^/(x^2^+y^2^+z^2^+1), b) shared environment c^2^ = y^2^/(x^2^+y^2^+z^2^+1), c) common unique environment e^2^ = z^2^/(x^2^+y^2^+z^2^+1), and d) age interval specific unique environment ε^2^ = 1/(x^2^+y^2^+z^2^+1). r_g_ denotes correlation between additive genetic effects and is set at 1 for MZ and 0.5 for DZ twins. r_c_ is the correlation between shared environmental effects and is assumed to be equal to 1 for both MZ and DZ twins.

In discrete-time survival analysis, unlike continuous-time survival analysis, time-to-event is measured discretely [22]. In this study, time-to-DP was categorized according to age at onset of DP. A model describing discrete-time frailty for DP in a twins setting with three age groups is presented in Figure 1. Three age intervals of age at onset of DP are represented by U_ti_, where *t* = 1, 2, 3 (corresponding to ≤49, 50–57, and 58–64 years) and *i* = 1, 2 (each indicating a twin in a pair). The outcome variable U_ti_ could obtain three values: value 0 (twin *i* is at risk but did not experience DP in age interval *t*); value 1 (twin *i* is at risk and experienced DP in age interval *t*); and value 9 (twin *i* is not at risk in age interval *t* due to the occurrence of an event other than DP (i.e., censored) or due to an earlier DP event). Latent frailty (F_i_) is common to all age intervals and is defined as a function of the event indicators (U_ti_) [4].

The number of age intervals included in the model depended on the number of available concordant twin pairs for DP in each age interval and for each zygosity, sex, and DP diagnosis group. At least one concordant twin pair should be available in each category. For analyses of specific diagnoses, three age groups (≤49, 50–57, and 58–64 years) could be created. However, there were too few concordant female and male twin pairs in each DP diagnosis group, and therefore the sex differences in liability to DP due to specific diagnoses could not be tested. Instead, we analyzed whether liability to DP differed between women and men when all DP diagnoses were pooled. For the computations, five age groups could be created: ≤45, 46–50, 51–55, 56–60, and 60–64 years.

Following traditional twin methods, variation in the observed time-to-DP (U_ti_) is assumed to arise from individual differences in common liability to DP (F_i_) caused by additive genetic effects (A), common environment (C), and unique environment (E). MZ twins are genetically identical at the sequence level, whereas DZ twins share on average half of all their segregating genes. Thus, the genetic correlation (r_g_) between MZ and DZ twins is set at 1.0 or 0.5. For OS twins, r_g_ is first estimated freely, which gives an indication of whether the same genes are expressed in women and men, and tested for statistical significance by fixing it at 0.5, which is the expected genetic correlation when no sex-specific genetic effects are present.

Both MZ and DZ twins are assumed to share to an equal degree their common environment, which includes the events (relevant to the granting of DP) that members of a twin pair experience together or are jointly exposed to. The common environment correlation (r_c_) is therefore set at 1.0 for both twin zygosities. Unique environment includes the individual influences that make twins dissimilar, and also measurement error. In this study, unique environment was modeled as one parameter unique to each twin for the whole follow-up period, and also as three or five parameters unique to each twin and each age interval (ε_t_). The effects of all genetic and environmental factors were assumed to be constant at different age intervals (see [4] for a detailed description of the model).

The genetic and environmental variance components can be estimated independently for women and for men. This allows us to test whether the magnitudes of genetic and environmental influences on common liability to DP differ between the sexes, i.e., whether there are any quantitative sex differences. Further, with inclusion of OS twins, we can also test whether the genes that contribute to liability to DP are the same in women and men, i.e., whether any qualitative sex differences are present.

Model estimation was performed using the maximum-likelihood approach in the Mplus statistical software [23]. Model fit was evaluated using the likelihood-ratio test, which compares the fit of the full discrete-time frailty model (including all three variance components A, C, and E that differed between females and males) with the fit of several constrained models (e.g., the AE model). The two-fold differences in log-likelihoods between the full and constrained models with a minus sign (−2LL) follow the χ^2^ distribution with Δ*df*. A significant difference indicates that a constrained model fits the data poorly, and thus that the eliminated parameters are important for the model. The parsimony of the model was assessed using Akaike's Information Criterion (AIC), where the lowest AIC value indicates the most parsimonious and best-explaining model.

## Results

### Descriptive statistics

**Figure 2:**
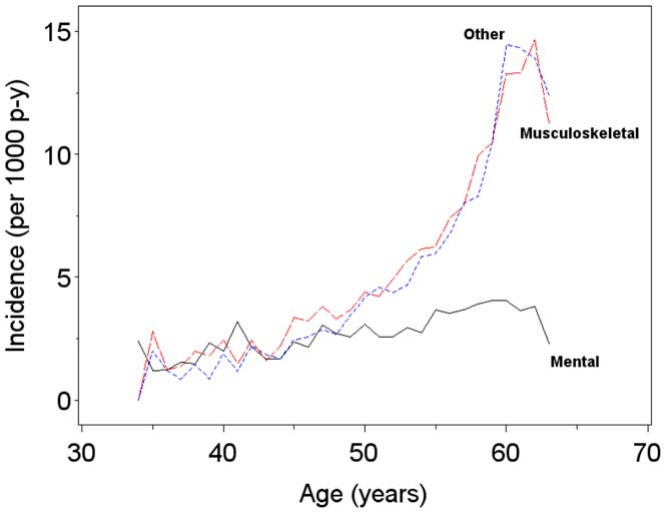
Incidence rates of disability pension per 1000 person-years in each diagnostic group.

**Figure 3:**
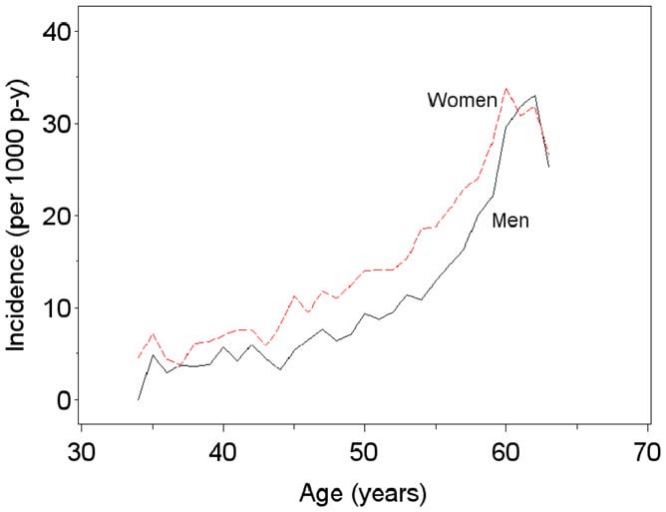
Incidence rates of disability pension per 1000 person-years for women and men.

During follow-up, a total of 7,669 individuals were granted DP (18.8% of the women and 14.1% of the men). There were 1,197 (8.1%) women and 677 (5.0%) men who were granted DP due to musculoskeletal diagnoses, and 572 (3.9%) women and 312 (2.3%) men who were granted DP due to mental diagnoses. The distribution of DPs granted in different diagnostic categories is presented in Table 1. The cumulative incidence of DP due to any diagnosis was 13.6% for MZ males, 13.5% for DZ males, 18.2% for MZ females, 19.1% for DZ females, 14.8% for OS males, and 18.9% for OS females. The incidence of DP in each diagnosis group was somewhat higher among women than men. Incidence rates of DP due to musculoskeletal and due to other diagnoses increased strongly with age (Figure 2). In contrast, DPs that were granted due to mental diagnoses had a more stable incidence at all ages. In all the diagnostic groups, lower incidence rates were observed after the age of 60 years. The incidence rate of DP due to any diagnosis was slightly higher among women than men up to the age of 62 years, when incidence rates became similar for both women and men (Figure 3).

**Table 1:** Cumulative incidence (%) of the granting of disability pension (DP) by sex, zygosity, and diagnosis group.

DP diagnoses	Men (same-sex pairs)		Women (same-sex pairs)		OS	
	MZ (n = 5094)	DZ (n = 8330)	MZ (n = 6002)	DZ (n = 8760)	Men (n = 9135)	Women (n = 9135)
All	13.55	13.54	18.19	19.09	14.83	18.96
Mental	1.98	2.53	4.00	3.79	2.52	4.05
Musculoskeletal	5.18	4.96	7.73	8.37	5.53	8.63
Others	6.38	6.05	6.46	6.93	4.53	5.00

Note. MZ: monozygotic twins, DZ: dizygotic same-sex twins, OS: dizygotic opposite-sex twins.

The numbers of concordant (either both healthy or both with DP) and discordant twin pairs for DP by diagnosis, sex, and zygosity group are shown in Table 2. The lowest concordance was observed among young men who were granted DP due to mental diagnoses. Within-pair correlations for DP due to each medical diagnosis are shown in Table 3. For all diagnoses, correlations for liability to DP within MZ twin pairs were approximately twice the size of those within DZ twin pairs, suggesting the importance of genetic factors. For example, for the youngest MZ twins with DP due to a mental diagnosis, the within-pair correlation was 0.64, but it was 0.38 for DZ twins. For DP due to mental diagnoses, the within-pair correlations decreased with increasing age, whereas for DP due to musculoskeletal and DP due to other diagnoses, the sizes of the correlations were similar to those for all age groups. Genetic influences on liability to DP due to any diagnosis were also suggested by the within-pair correlations calculated for women and men separately (Table 4). In addition, correlations within OS twin pairs were lower than within DZ twin pairs, suggesting that qualitative sex differences may be present.

**Table 2:** Numbers (%) of concordant and discordant twin pairs in different disability pension (DP) diagnostic groups, by sex and zygosity.

DP diagnoses	Men						Women						Women/Men			
	MZ (n = 2547 pairs)			DZ (n = 4164 pairs)			MZ (n = 3001 pairs)			DZ (n = 4380 pairs)			OS (n = 9135 pairs)			
	C+	C−	D	C+	C−	D	C+	C−	D	C+	C−	D	C+	C−	D^a^	D^b^
All	111 (4.36)	1968 (77.27)	468 (18.37)	121 (2.91)	3157 (75.80)	886 (21.27)	190 (6.33)	2099 (69.94)	712 (23.73)	229 (5.23)	2937 (67.05)	1214 (27.72)	355 (3.89)	6403 (70.09)	1377 (15.07)	1000 (10.95)
Mental	6 (0.24)	2452 (96.27)	89 (3.49)	8 (0.19)	3961 (95.12)	195 (4.68)	27 (0.90)	2788 (92.90)	186 (6.20)	19 (0.43)	4067 (92.85)	294 (6.71)	18 (0.20)	8553 (93.63)	369 (4.04)	195 (2.13)
Musculoskeletal	25 (0.98)	2308 (90.62)	214 (8.40)	25 (0.60)	3776 (90.68)	363 (8.72)	50 (1.67)	2587 (86.20)	364 (12.13)	53 (1.21)	3700 (84.47)	627 (14.32)	72 (0.79)	7914 (86.63)	732 (8.01)	417 (4.56)
Others	35 (1.37)	2257 (88.61)	255 (10.01)	24 (0.58)	3684 (88.47)	456 (10.95)	23 (0.77)	2636 (87.84)	342 (11.39)	29 (0.66)	3802 (86.80)	549 (12.53)	45 (0.49)	7986 (87.42)	580 (6.35)	524 (5.74)

Note. C+ concordant for DP diagnosis, C− concordant for no DP, D discordant;

a woman on DP,

b man on DP.

**Table 3:** Within-pair correlations (95% CI) for liability to disability pension (DP) for the main DP diagnostic groups, by zygosity pooled over the sexes.

DP diagnoses	MZ pairs (n = 5548)			DZ pairs (n = 17679)		
	≤49 (n = 165)	50–57 (n = 313)	58–64 (n = 405)	≤49 (n = 586)	50–57 (n = 1097)	58–64 (n = 1337)
Mental	0.64 (0.54–0.74)	0.49 (0.37–0.60)	0.42 (0.25–0.59)	0.38 (0.25–0.50)	0.28 (0.16–0.40)	0.22 (0.04–0.41)
Musculoskeletal	0.57 (0.47–0.67)	0.38 (0.29–0.47)	0.43 (0.34–0.51)	0.21 (0.07–0.34)	0.24 (0.16–0.33)	0.22 (0.14–0.30)
Others	0.51 (0.39–0.63)	0.35 (0.25–0.45)	0.37 (0.28–0.46)	0.19 (0.04–0.34)	0.06 (−0.05–0.11)	0.23 (0.15–0.31)

Note. Correlations were calculated using a 3-group categorization of age. DZ twins include both same-sexed and opposite-sexed twins.

**Table 4:** Within-pair correlations for liability (95% CI) to disability pension (DP) among women and men, by age group and zygosity.

	< = 45 (n = 279)		46–50 (n = 526)		51–55 (n = 868)		56–60 (n = 1217)		61–64 (n = 1314)	
	MZ	DZ	MZ	DZ	MZ	DZ	MZ	DZ	MZ	DZ
All DP diagnoses										
Men	0.51 (0.33–0.69)	0.36 (0.16–0.55)	0.43 (0.26–0.59)	0.21 (0.07–0.36)	0.44 (0.32–0.56)	0.25 (0.13–0.36)	0.42 (0.32–0.51)	0.22 (0.12–0.31)	0.38 (0.29–0.47)	0.29 (0.21–0.37)
Women	0.66 (0.48–0.83)	0.50 (0.38–0.62)	0.37 (0.24–0.50)	0.21 (0.09–0.33)	0.36 (0.27–0.46)	0.28 (0.20–0.37)	0.32 (0.24–0.41)	0.21 (0.14–0.29)	0.25 (0.15–0.34)	0.19 (0.12–0.27)
OS	0.27 (0.15–0.40)		0.20 (0.12–0.29)		0.15 (0.08–0.22)		0.18 (0.12–0.23)		0.20 (0.14–0.25)	

Note. Correlations were calculated using a 5-group categorization of age. OS = opposite sexed twin pairs.

### Model-fitting results

The model-fitting analyses of pooled data for women and men based on the two zygosity groups showed that the genetic and unique environmental factors (AE) model best explained the variance in DP due to any diagnosis, and also in DP in each specific diagnostic group. The standardized parameter estimates of the AE model are presented in Table 5. Genetic effects (a^2^) explained 49% (95% CI: 39–59) of the variance in DP due to mental, 35% (95% CI: 29–41) due to musculoskeletal, and 27% (95% CI: 20–33) due to all other diagnoses. Unique environment (e^2^) played a minor role in liability to DP. Factors that were specific to each age (ε^2^) explained 45% (95% CI: 22–69), 62% (95% CI: 54–69), and 66% (95% CI: 55–77) of the variance in liability to DP due to mental, musculoskeletal, and all other diagnoses, respectively.

**Table 5:** Estimates of variance components of the best-fitting model (AE) for liability to disability pension (DP) due to all diagnoses, and DP by diagnostic group.

DP diagnoses	Variance components (95% CI)		
	a^2^	e^2^	ε^2^
All	0.33 (0.28–0.38)	0.05 (0.00–0.18)	0.62 (0.46–0.77)
Mental	0.49 (0.39–0.59)	0.06 (0.00–0.24)	0.45 (0.22–0.69)
Musculoskeletal	0.35 (0.29–0.41)	0.03 (0.00–0.08)	0.62 (0.54–0.69)
Others	0.27 (0.20–0.33)	0.07 (0.00–0.16)	0.66 (0.55–0.77)

Model-comparison results for sex differences in liability to DP due to any diagnosis are presented in Table 6. The model-fitting analyses started by fitting a general ACE model with different parameters for women and men, and also an estimated genetic correlation (which allows for qualitative sex differences). The sex-limitation ACE model was used as the main model with which all the other constrained models were compared. The importance of shared environment for liability to DP was tested by setting the value of the C component at 0, for both women and men. Compared with the main ACE model, this resulted in a non-significant deterioration in model fit (Model II, r_g_ free: Δχ^2^ = 0.48, Δdf = 2, p = 0.79) and a decrease in the AIC value. However, omitting component A resulted in a model with a much poorer fit (Model III, r_g_ free: Δχ^2^ = 68.24, Δdf = 2, p<0.001), and also an increase in the AIC value. Further, quantitative sex differences were tested by fitting an AE model where genetic and environmental parameters were constrained to be equal for women and men. This resulted in a non-significant deterioration in model fit (Model IV: Δχ^2^ = 0.99, Δdf = 4, p = 0.91), and also a decreased AIC value. Finally, to examine whether genetic effects differed qualitatively between the sexes, the genetic correlation for OS twin pairs (r_g_) was set at 0.5. This did not produce a significantly poorer model fit to data (Model V: Δχ^2^ = 6.78, Δdf = 5, p = 0.24), but there was an increased AIC value compared with Model IV. Hence, the AE model with equal parameters for women and men, but with freely estimated genetic correlation (Model IV), provided the best balance of parsimony and fit, as indicated by the lowest AIC value. Estimates of the variance components of the best-fitting model are presented in Figure 3. For both women and men, genetic effects common to all ages explained one-third, and age-specific factors almost two-thirds, of the total variance in liability to DP.

**Table 6:** Model-comparison results and variance component estimates (95% CI) for liability to disability pension (DP) (all diagnoses) among men and women.

Parameters	Model				
	I. ACE, r_g_ free	II. AE, r_g_ free	III. CE, r_g_ free	IV. AE^a^, r_g_ free	V. AE^a^, r_g_ = 0.5
a^2^_m_	0.32 (0.25–0.38)	0.31 (0.25–0.37)	-	**0.30 (0.25–0.35)**	0.28 (0.24–0.33)
c^2^_m_	0.00 (0.0–0.0)	-	0.16 (0.11–0.21)	**-**	-
e^2^_m_	0.10 (0.00–0.22)	0.07 (0.00–0.16)	0.17 (0.00–0.40)	**0.09 (0.00–0.21)**	0.10 (0.00–0.22)
ε^2^_m_	0.59 (0.44–0.74)	0.62 (0.50–0.74)	0.67 (0.42–0.93)	**0.62 (0.45–0.78)**	0.62 (0.47–0.78)
a^2^_f_	0.30 (0.00–0.64)	0.29 (0.19–0.39)		**0.30 (0.25–0.35)**	0.28 (0.24–0.33)
c^2^_f_	0.00 (−0.26–0.26)	-	0.26 (0.04–0.48)	**-**	-
e^2^_f_	0.14 (0.00–0.42)	0.10 (0.00–0.42)	0.28 (0.00–0.87)	**0.09 (0.00–0.21)**	0.10 (0.00–0.22)
ε^2^_f_	0.57 (0.20–0.93)	0.61 (0.21–1.00)	0.65 (0.31–0.99)	**0.62 (0.45–0.78)**	0.62 (0.47–0.78)
Genetic correlation, r_g_	0.36 (0.13–0.58)	0.36 (0.13–0.58)	0.10 (0.10–0.10)	**0.36 (0.25–0.48)**	0.5
Δχ2	-	0.48	68.24	**0.99**	6.78
Δdf	-	2	2	**4**	5
p	-	0.79	<0.001	**0.91**	0.24
ΔAIC	-	−3.52	64.24	**−7.01**	−3.22

Note.

a Equal parameters for men and women; best-fitting model in bold. Variance components included in the models are denoted by A, C, and/or E, whereas estimated coefficients are denoted by a^2^, c^2^, e^2^, and ε^2^. See the main text for further explanation of the parameters and model-fitting procedure.

## Discussion

The present study aimed to investigate the effects of genetic and environmental factors on liability to DP in a population-based cohort of 46,454 twins initially eligible for DP and followed for 16 years. Also, sex differences in liability to DP were tested. Moderate to large genetic contributions were found for liability to DP, irrespective of the age at which DP was granted. At least equally important were the environmental factors influencing the trend in DP in each specific age group. No quantitative sex differences were found that is, the amount of variance explained by genetic and environmental factors for liability to DP was similar among both women and men. However, the results for DP, irrespective of underlying diagnosis, suggested that the genes operating in the pathways leading to DP may differ between women and men; that is, qualitative sex differences might be present.

Results regarding genetic liability to DP due to different diagnoses are consistent with the findings of the previously mentioned study of the Finnish population [4], although genetic liability in the present study was slightly higher for DPs due to mental diagnoses, and somewhat lower for DPs due to musculoskeletal diagnoses, compared with the Finnish results. The slightly different results obtained in these two studies may reflect random variation, or may depend on the age of the participants and thereby the length of follow-up. In the study of Finnish twins, a 30-year follow-up of individuals regarding incidence of DP began in 1975 and the youngest participants were then in their early 20 s, whereas the current study consisted in a 16-year follow-up of individuals who were 35 years or older at inclusion in 1993. Accordingly, participants were representative of all age groups in the Finnish investigation, whereas, for the present study, younger individuals were not included.

Genetic influences on liability to DP can be explained in several ways. As reported in previous twin studies, musculoskeletal diseases and mental disorders were at least partly heritable [6], [8], [9]. Thus, genetic contributions to liability to DP may reflect genetic susceptibility to a specific disease, which affects work capacity and leads to DP. However, given that the data analyses were performed for broad diagnostic categories, such as mental disorders, conditions with high heritability (e.g., schizophrenia and bipolar disease) will be present alongside conditions with lower heritability (e.g., depression and anxiety) in one and the same category. Also, a twin pair was considered as concordant if, for example, one twin was granted DP due to schizophrenia and the other twin was granted DP due to depression. An example of a discordant twin pair is a pair of twins with schizophrenia, in which one is granted DP and the other twin commits suicide soon after the onset of disease. Thus, the genetic liability to DP only partially reflects the genetics of the underlying conditions. Also, genetic factors were shown to account for a substantial amount of variance in functional ability [11], which may affect genetic liability to DP. Further, previous studies of DP have related several biological and early childhood factors (e.g., abnormal birth weight, chronic childhood disease, or early deviant behavior) to a higher risk of future DP [3], [24]. These factors have been shown to be partly heritable in several studies and could possibly mediate the genetic influences on liability to DP.

The magnitudes of genetic and environmental influences on liability to DP were found to be similar among women and men. That is, there were no sex differences in the amount of variance explained by genetic and environmental factors for DP. However, the results suggest that the sets of genes contributing to the variation in liability to DP may not be identical for both sexes. That is, the heritable factors that affect the development of DP seem to be different in women and men. In addition to biologic dissimilarities between the sexes, these factors may also include, for example, different susceptibility to a disease, differential response to childhood adverse experiences, or variation in functional abilities (e.g., strength or agility). No previous studies have investigated this issue and, therefore, the current findings cannot be compared with those from other populations. However, genetic sex differences have been reported earlier in a few twin studies of psychopathology. For example, sex-specific genetic factors have been reported to be influential on depression [8], [25], [26] and alcohol-related diagnoses [27]. Genetic heterogeneity between females and males was also demonstrated for rheumatoid arthritis [28]. In general, only a few studies include OS twin pairs and, therefore, the research on sex-specific genetic factors for different health outcomes is scarce.

Findings on sex-specific effects on liability to DP may also reflect sex differences with regard to each diagnosis. Because there were too few concordant twin pairs in each age group in our study, this hypothesis could not be tested. Thus, we cannot rule out the possibility that our findings of sex-specific genetic effects may depend, for example, on a different distribution of diseases between women and men. It is also possible that qualitative genetic differences between the sexes in liability to DP may be primarily due to the sex differences in, for example, psychiatric diagnoses, whereas similar sets of genes may operate in liability to DP due to musculoskeletal diagnoses. Worth noting is that specific diagnosis categories may have different development patterns in women and men. For example, schizophrenia and related psychotic disorders with a peak onset in young adulthood (<35 years) do not show major gender differences [29]. On the other hand, substance abuse and dependence often has an early onset in adolescence and is much more common in men, whereas depression is a later onset condition and is more common in women [29].

In line with the results of the Finnish study [4], it was found that the effect of genetic factors diminished over time, primarily with increasing age. That is, as age increases, environmental factors become more important for liability to DP. Previous research suggests that increases in variance with age may be due to an increase in environmental variance, as people accumulate their exposures and experiences over the life-span [30], [31]. Studies of the influences of genetic and environmental factors on other health outcomes (e.g., self-rated health or physical functioning) have shown similar results [32], [33], [34].

The incidence rate of DP was lower in the 60-years-and-older age group than in younger age groups. This may reflect the increasing number of old-age pensioners in this age group, and also the fact that most of the individuals with health problems have already been granted a DP. Alternatively, individuals with health problems that first arise at later ages may continue working and/or be on sick-leave until old-age retirement.

The study has several strengths. The sample was large, population-based, and was followed for 16 years. Because all data were obtained by linking a number of national registers, there were no information or response biases. Information on all individuals was detailed and of high quality, with no loss to follow-up. Findings of potential sex differences for genetic liability to DP are, to our knowledge, reported here for the first time. The main limitation of the study relates to the relatively small number of DPs granted during the follow-up period. Since the earliest age of follow-up was around the mid-30 s, the processes leading to DP in young adults could not be investigated, and those with DP by the age of 35 were not included in the analyses. Despite the large sample and a reasonable follow-up time, the number of DPs granted was too small to investigate the genetic liability to DP of women and men in each DP diagnosis group. Therefore, further studies should be performed to examine this issue further by following individuals for a longer time, e.g. from young adulthood to retirement. Also, the liability to DP due to different diagnoses could be studied by pooling data on DP from several countries that have similar DP granting systems to Sweden.

Another limitation concerns possible selection bias. For the data analyses, twins with unknown zygosity or with missing follow-up information on their co-twin were excluded from the cohort. The cumulative incidence of DP among excluded men was higher than among men in the total cohort (18.1% vs. 14.1%). For women, there was only a marginal difference between the incidence of DP in the excluded twins and that in the total cohort (18.8% vs. 18.0%). This suggests that more severe diseases or diseases of higher heritability may have been more prevalent among the excluded male twins. Thus, the genetic liability to DP reported in this study may be slightly underestimated. As suggested by the decreasing influence of genetic factors on liability to DP with increasing age, the genetic liability may be underestimated primarily at later ages. That is, individuals with higher genetic liability to DP may have a more severe disease and be granted DP earlier than others.

The findings of this study are similar to those from the previous analyses of Finnish data, and suggest that genetic effects are important for liability to DP due to different diagnoses. Further, genetic effects contributing to liability to DP seem to be different among women and men. Hence, pathways leading to DP seem to differ for women and men, which should be borne in mind when interventions to prevent DP are planned. However, further large and prospective studies are needed to be able to identify specific factors that mediate genetic effects for each diagnosis and sex group, so as to increase our understanding of the obviously complex mechanisms involved in trends in DP.
